# Supplementary material for: The preeminence of ethnic diversity in scientific collaboration
Source: Nat Commun. 2018 Dec 4;9:5163. doi: 10.1038/s41467-018-07634-8 (PMC6279741; doi:10.1038/s41467-018-07634-8)
Supplement: Supplementary file 1 — Supplementary Information [file 41467_2018_7634_MOESM1_ESM.pdf]

Supplementary Materials for

The Preeminence of Ethnic Diversity in Scientific Collaboration

Bedoor K AlShebli*, Talal Rahwan*, Wei Lee Woon*

*Joint corresponding authors. E-mail: bedoor@deeplearn.net; talal.rahwan@nyu.edu; wlwoon@deeplearn.net

Supplementary Figures

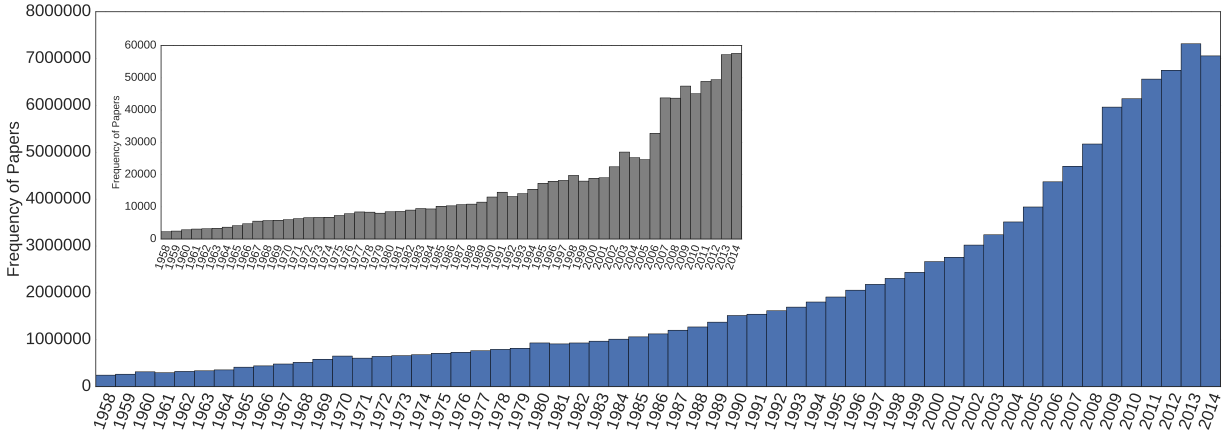

Supplementary Figure 1: The distribution of papers published each year in the Microsoft Academic Graph corpus. The inset shows the distribution of our sample set, consisting of 1,045,401 papers.

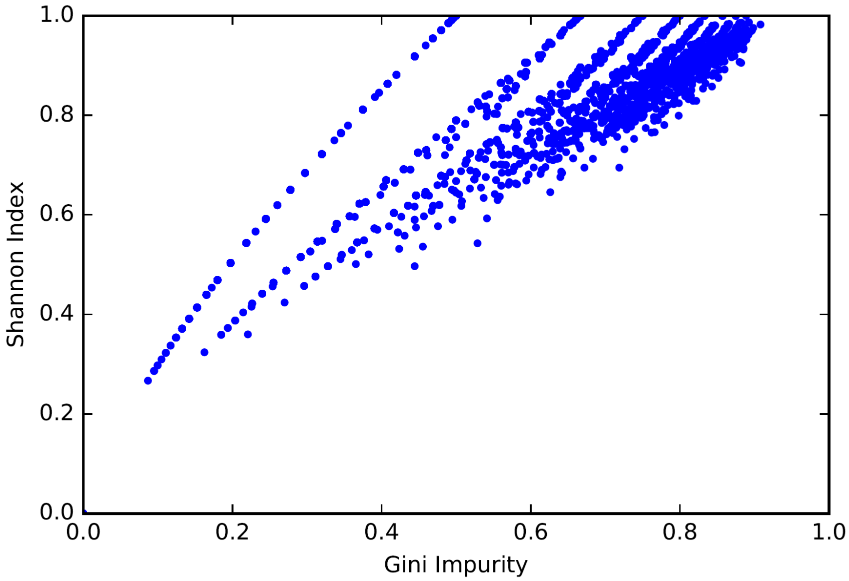

Supplementary Figure 2: For every group of scientists that coauthored a paper in the Microsoft Academic Graph dataset, we measured the ethnic diversity using both the Shannon entropy and the Gini index. The two are plotted against each other, showing a clear correlation (Pearson's $r = 0.93$ and $p < 0.0001$).

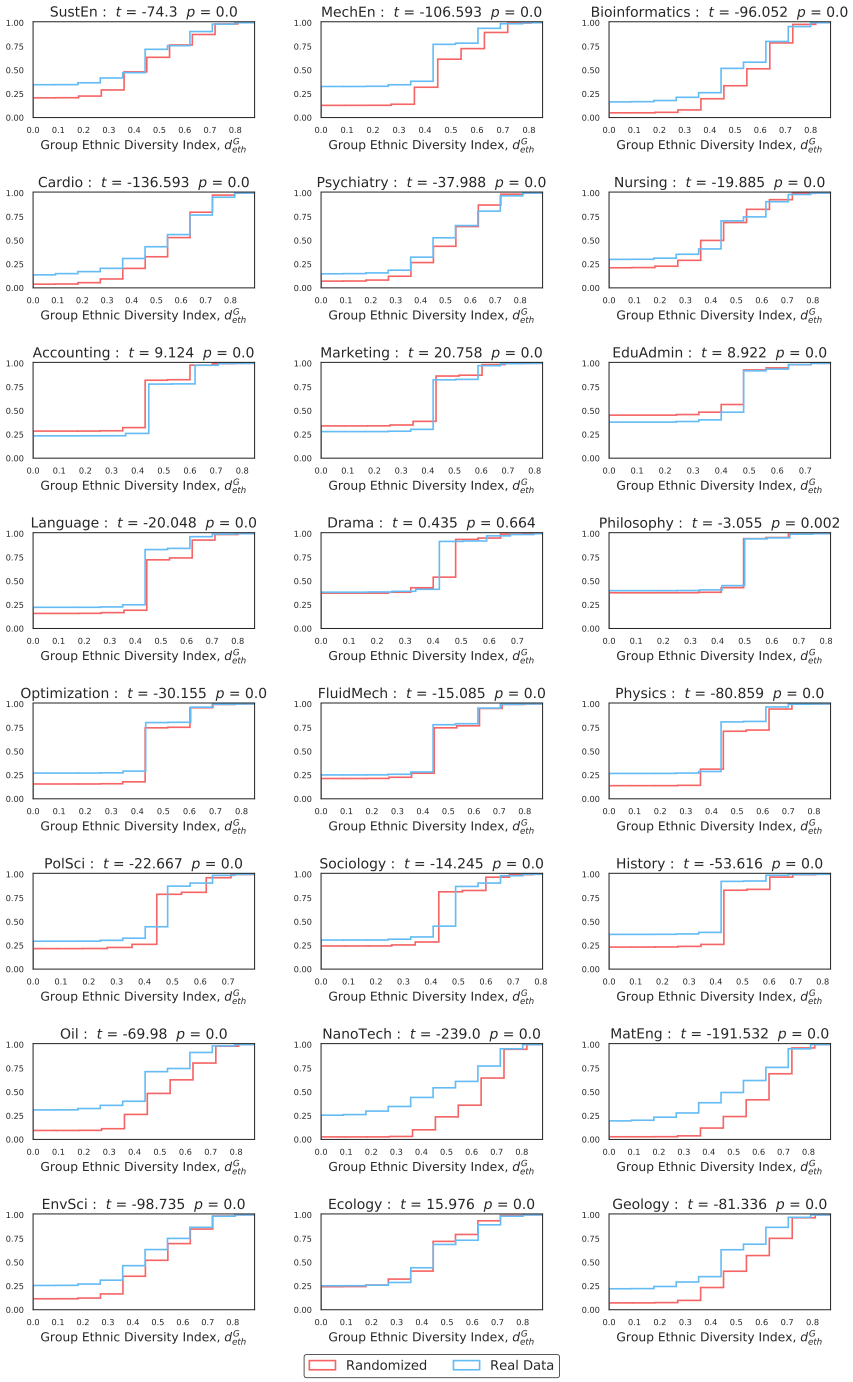

Supplementary Figure 3: Cumulative distribution function (CDF) of group **ethnic diversity**, d^G_{eth}, for the real and randomized data. In all 24 subfields, groups with low d^G_{eth} are more common in reality than expected by random chance, highlighting the fact that homophily does indeed exist in academia. For all subfields, the difference between the two datasets is statistically significant ($p \ll .05$).

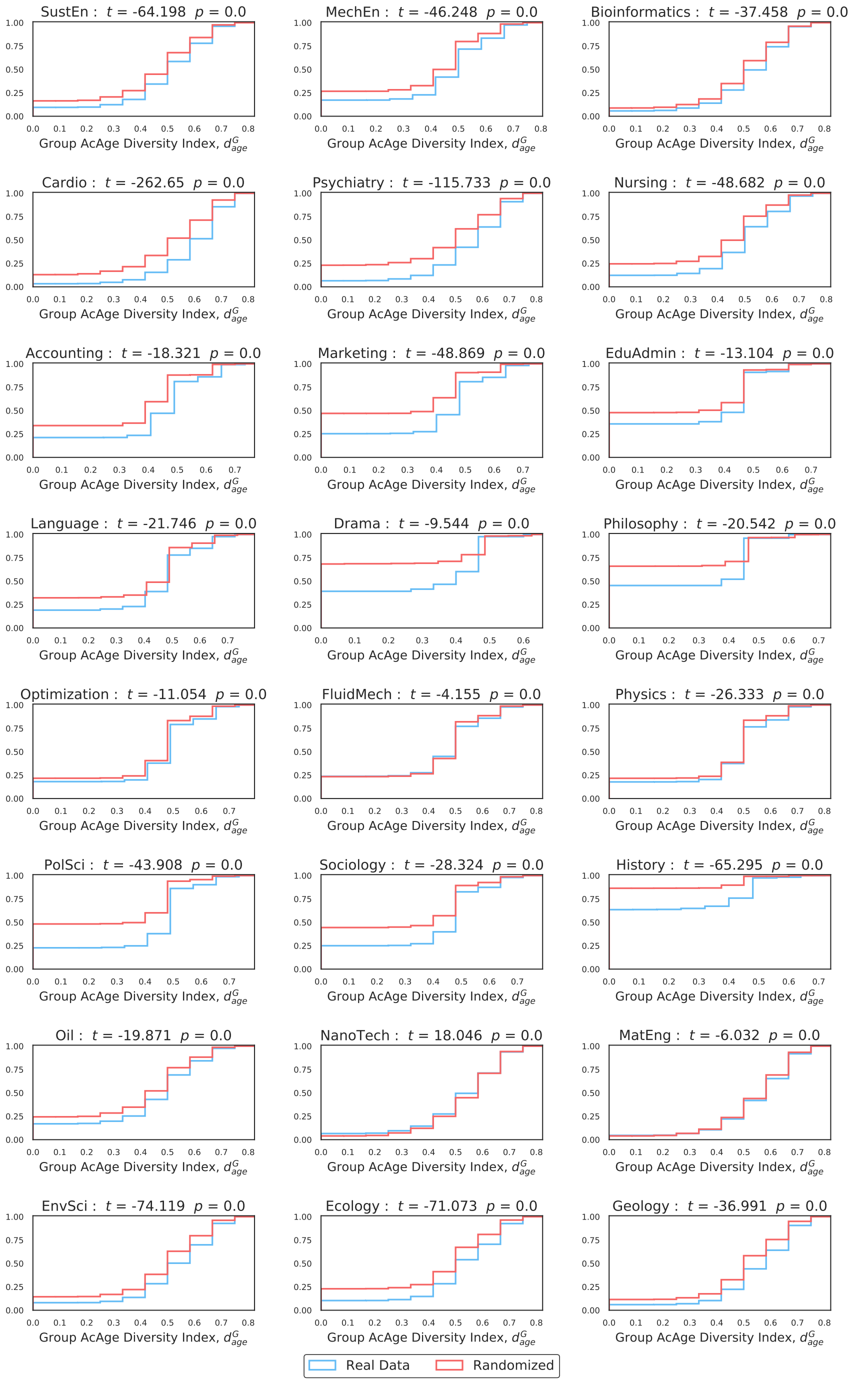

Supplementary Figure 4: Cumulative distribution function (CDF) of group **age diversity**, d^G_{age}, for the real and randomized data. In all 24 subfields, groups with low d^G_{age} are more common in reality than expected by random chance, highlighting the fact that homophily does indeed exist in academia. For all subfields, the difference between the two datasets is statistically significant ($p \ll .05$).

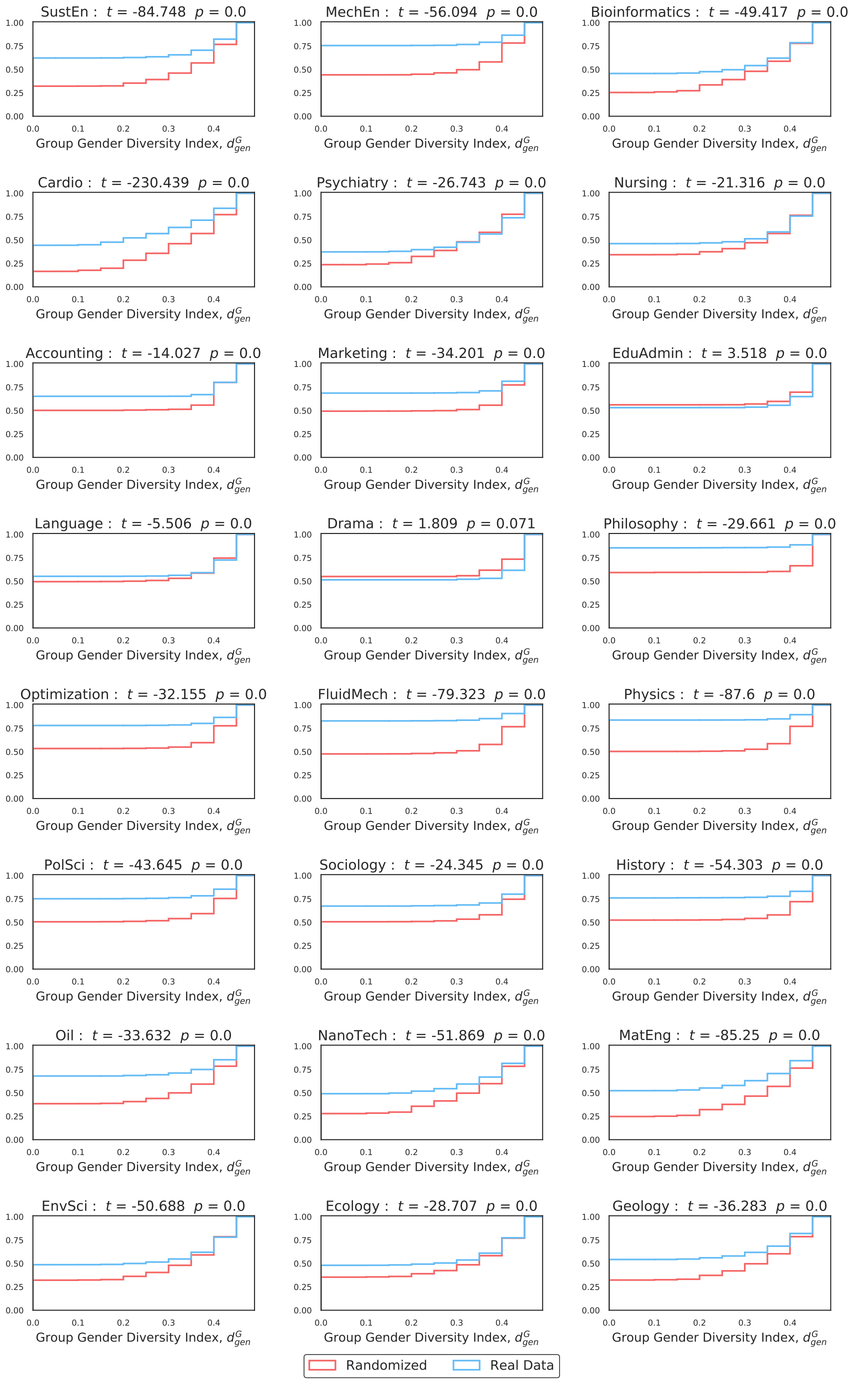

Supplementary Figure 5: Cumulative distribution function (CDF) of group **gender diversity**, d^G_{gen}, for the real and randomized data. In all 24 subfields, groups with low d^G_{gen} are more common in reality than expected by random chance, highlighting the fact that homophily does indeed exist in academia. For all subfields, the difference between the two datasets is statistically significant ($p \ll .05$).

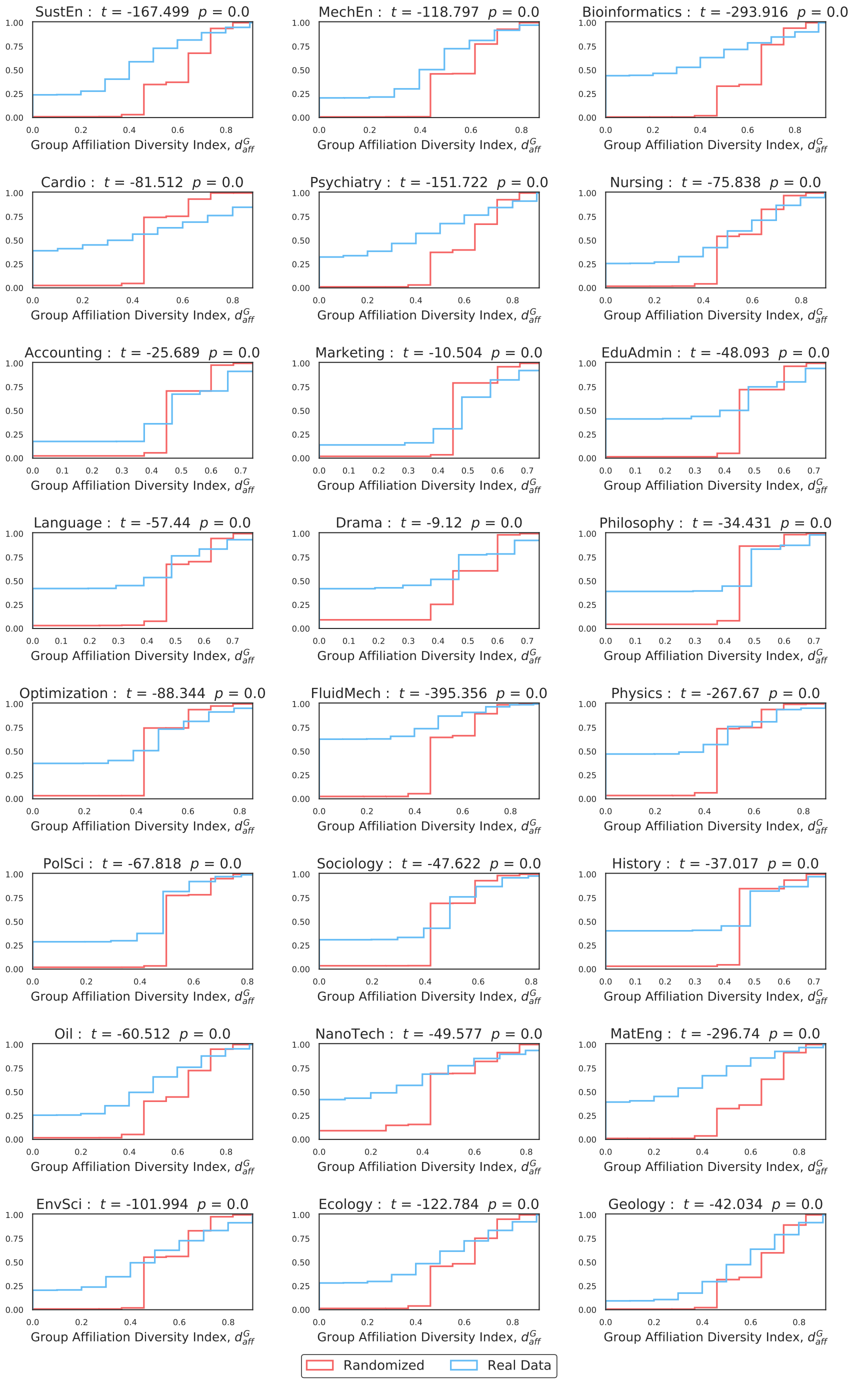

Supplementary Figure 6: Cumulative distribution function (CDF) of group **affiliation diversity**, d^G_{aff}, for the real and randomized data. In all 24 subfields, groups with low d^G_{aff} are more common in reality than expected by random chance, highlighting the fact that homophily does indeed exist in academia. For all subfields, the difference between the two datasets is statistically significant ($p \ll .05$).

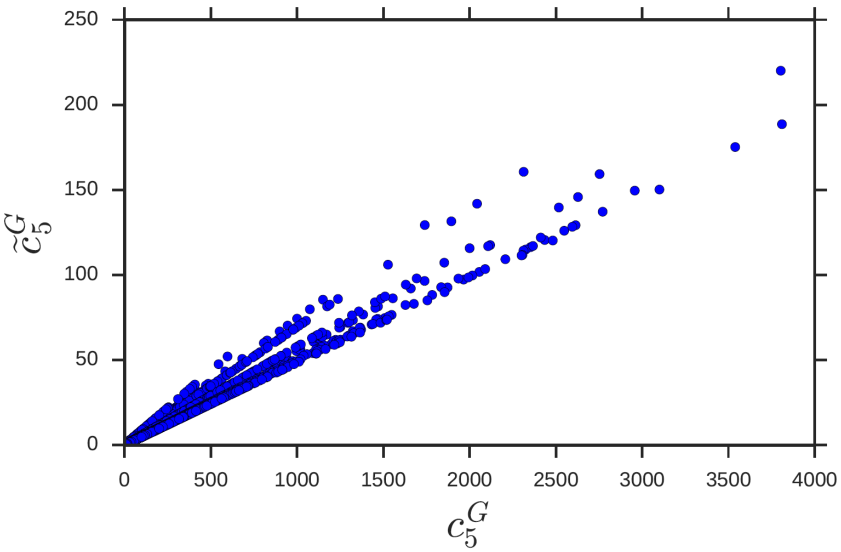

Supplementary Figure 7: Using 500,000 papers sampled from the entire MAG dataset, we compare c_5^G with an alternative, normalized measure of impact, denoted as $\widetilde{c}_5^G$ (see Supplementary Note 4 for more details). As can be seen, the two are very strongly correlated (over the entire dataset, Pearson's $r = 0.965$ and $p < 0.0001$).

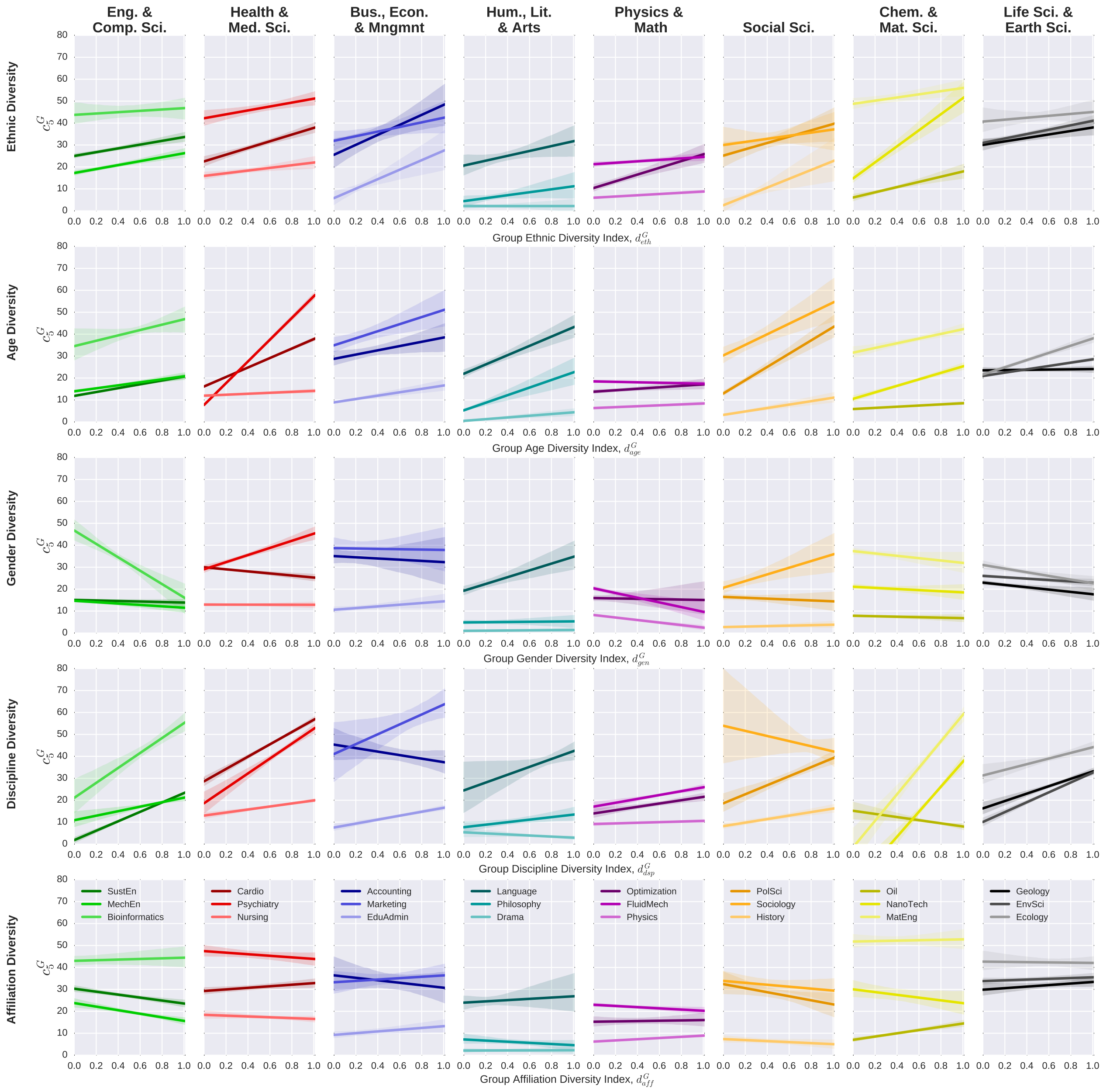

Supplementary Figure 8: Group diversity against c^G_5 in each of the 24 subfields, which are grouped into the 8 main fields in Google Scholar. In the case of group *ethnic* diversity and group *age* diversity, every significant correlation with c^G_5 is positive, and nearly all correlations were significant. This, however, does not hold for the remaining group diversity indices (see the corresponding p-values in Supplementary Table 2).

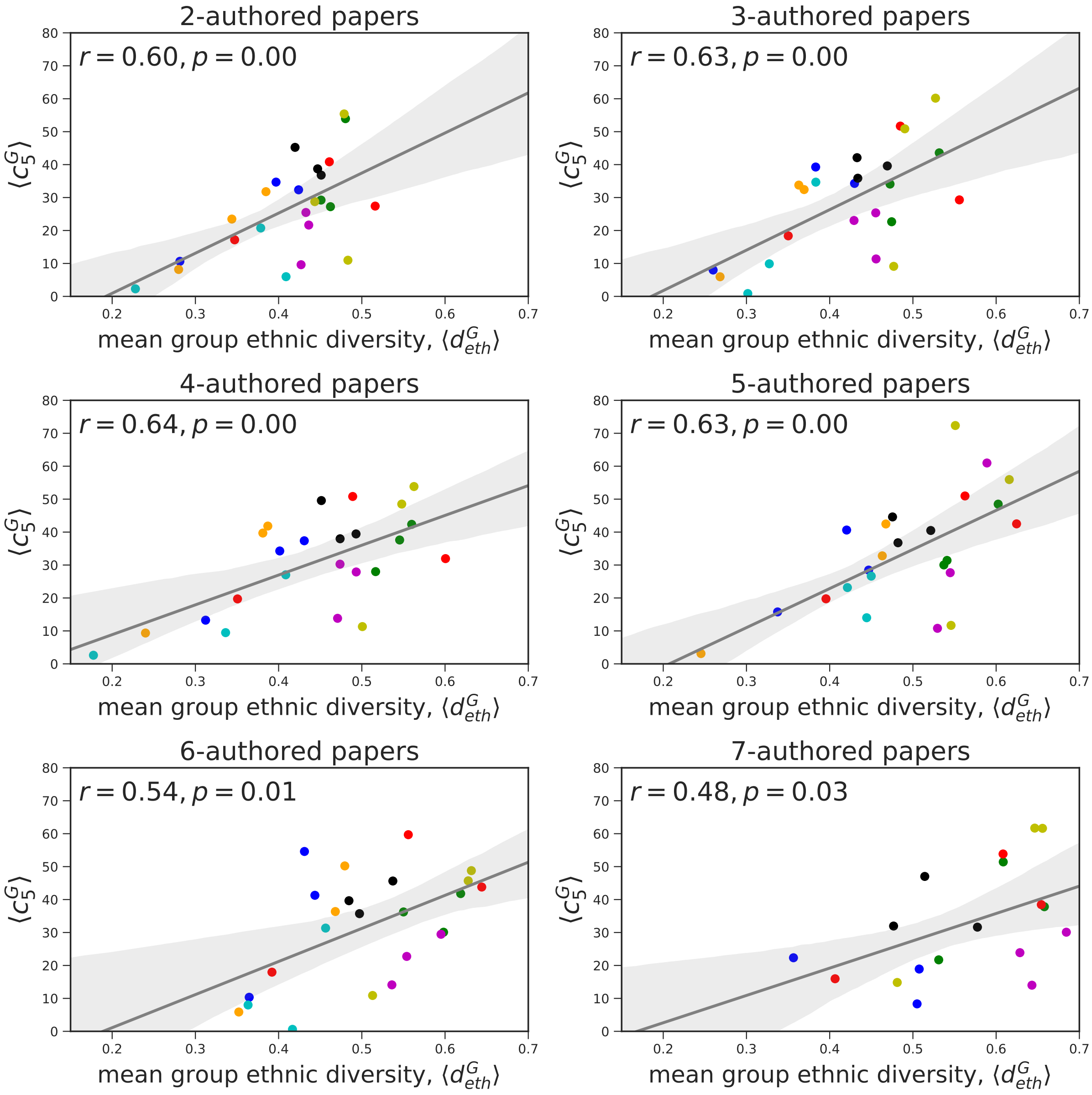

Supplementary Figure 9: Mean group ethnic diversity against mean paper impact in each subfield while controlling for the number of authors. In each subplot, the color indicates the main field, while the solid line and the shaded area represent the regression line and the 95% confidence interval, respectively. Each regression has also been annotated with the corresponding Pearson's r and p values. For each subfield, the subplots depict the mean group ethnic diversity, $\langle d^G_{eth} \rangle$, against the mean five-year citation count, $\langle c^G_5 \rangle$, taken over papers in that subfield while controlling for the number of authors in each paper.

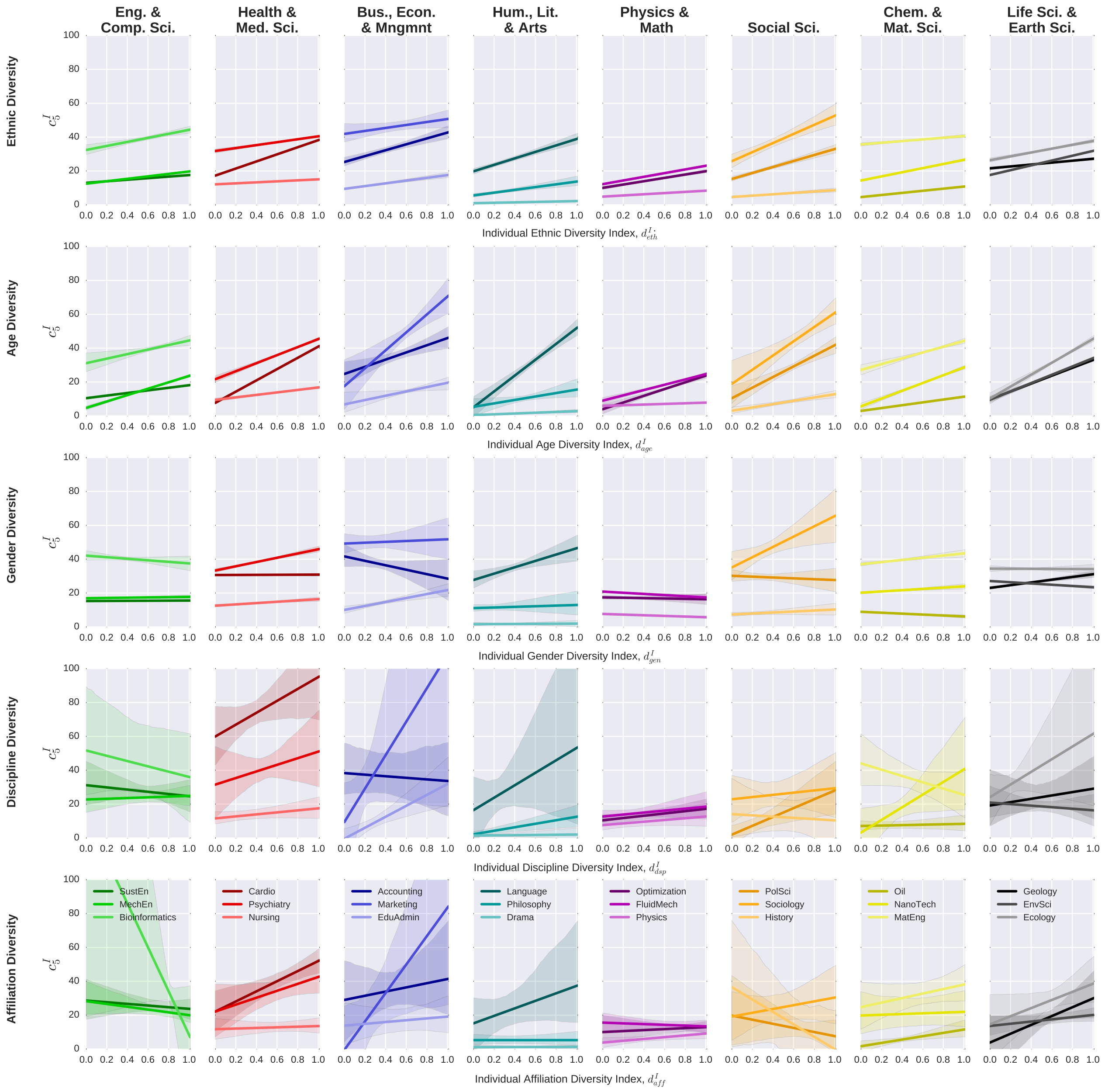

Supplementary Figure 10: Individual diversity against c_5^I in each of the 24 subfields, which are grouped into the 8 main fields in Google Scholar. In the case of individual *ethnic* diversity, every correlation with c_5^I is significantly positive. The same holds for individual *age* diversity, with the exception of two subfields (Philosophy and Drama) for which the correlations are positive but not significant. This, however, does not hold for the remaining individual diversity indices (see the corresponding p-values in Supplementary Table 4).

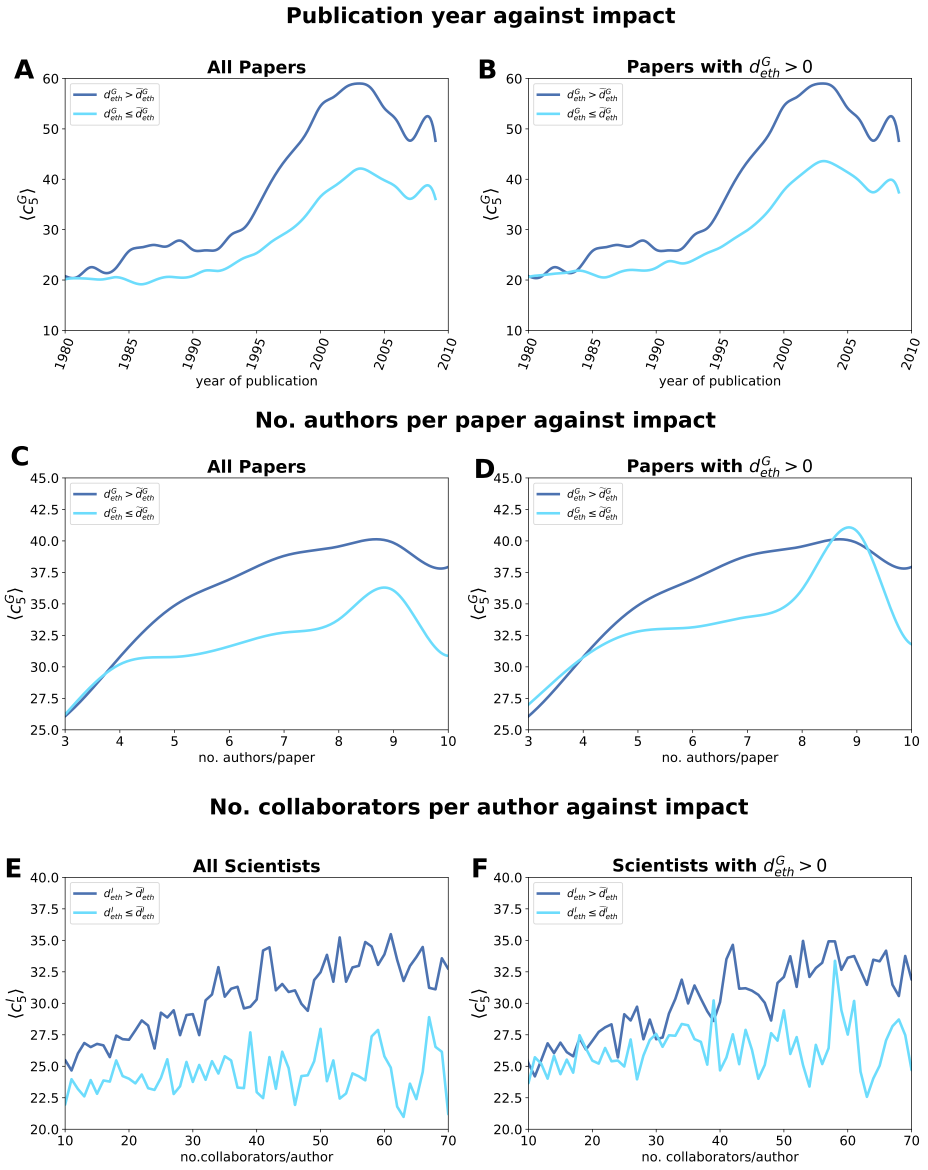

Supplementary Figure 11: Excluding papers and scientists with no ethnic diversity. **(A)** Comparison between "diverse" papers (where $d^G_{eth} > \widetilde{d}^G_{eth}$) against "non-diverse" ones (where $d^G_{eth} \leq \widetilde{d}^G_{eth}$) given different publication years. **(B)** Same analysis as in (A), but after excluding all papers for which $d^G_{eth} = 0$. **(C)** Comparison between diverse and non-diverse papers given different numbers of authors. **(D)** Same analysis as in (C), but after excluding all papers for which $d^G_{eth} = 0$. **(E)** Comparison between diverse scientists (whose $d^I_{eth} > \widetilde{d}^I_{eth}$) against non-diverse ones (whose $d^I_{eth} \leq \widetilde{d}^I_{eth}$) given different numbers of collaborators. **(F)** Same analysis as in (E), but after excluding all scientists for which $d^I_{eth} = 0$.

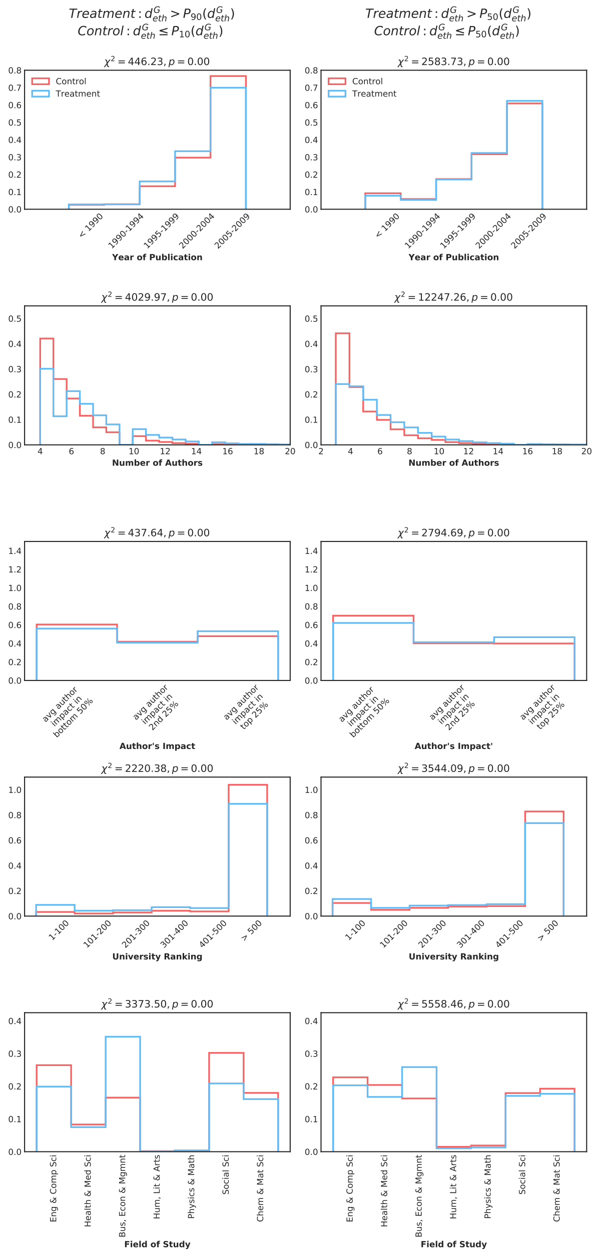

Supplementary Figure 12: The distribution (before matching) of the treatment and control groups for each confounding factor in the coarsened exact matching (CEM) when studying the causal effect of **group** ethnic diversity on scientific impact. The treatment and the control sets consist of papers for which $d^G_{eth} > P_{100-i}\left(d^G_{eth}\right)$ and $d^G_{eth} \leq P_i\left(d^G_{eth}\right)$, respectively, where $P_i\left(d^G_{eth}\right)$ denotes the ith percentile of d^G_{eth}. In our study, we repeated this process using $i = 10, 20, 30, 40, 50$, but the figure only depicts the distributions for the cases where $i = 10$ (left column) and where $i = 50$ (right column). For the sub-figures in the second row (number of authors), the x-axes have been truncated to exclude outliers. Each subfigure has been annotated with its corresponding Chi-Squared test results, all of which were significant ($p < 0.0001$).

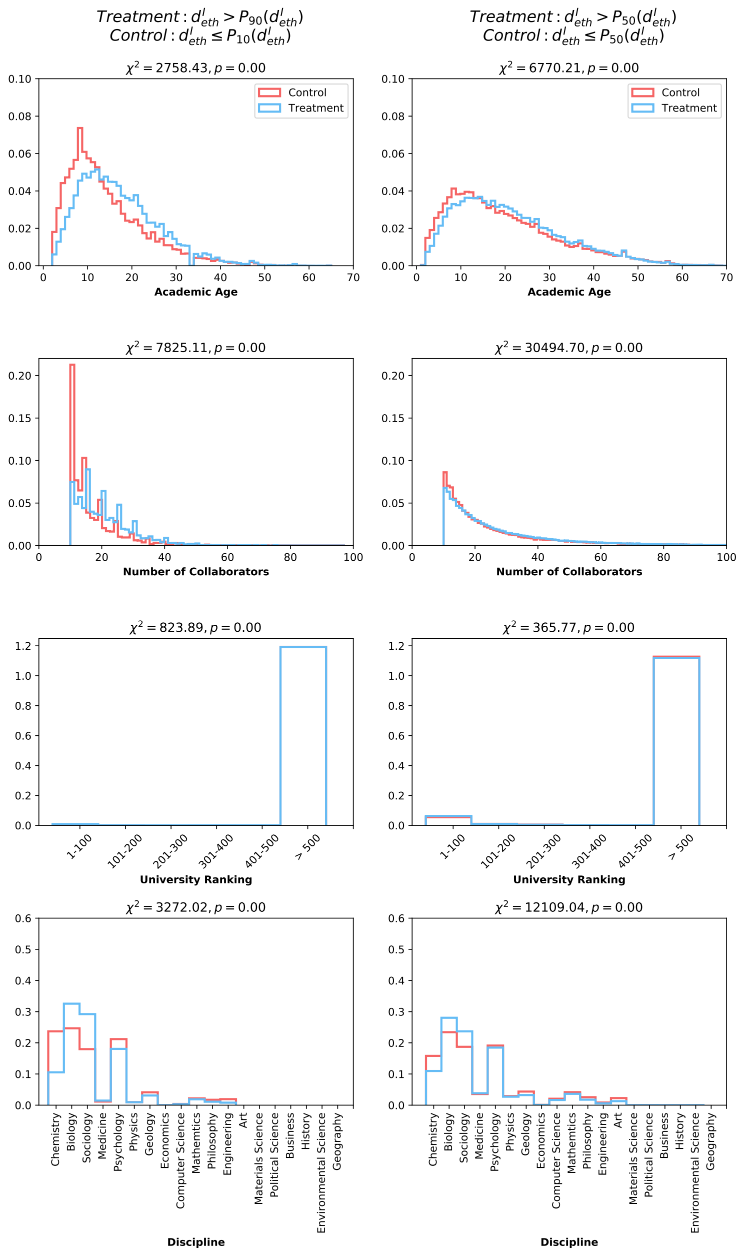

Supplementary Figure 13: The distribution (before matching) of the treatment and control groups for each confounding factor in the coarsened exact matching (CEM) when studying the causal effect of **individual** ethnic diversity on scientific impact. The treatment and the control sets consist of scientists for which $d^I_{eth} > P_{100-i}\left(d^I_{eth}\right)$ and $d^I_{eth} \leq P_i\left(d^I_{eth}\right)$, respectively, where $P_i\left(d^I_{eth}\right)$ denotes the ith percentile of d^I_{eth}. In our study, we repeated this process using $i = 10, 20, 30, 40, 50$, but the figure only depicts the distributions for the cases where $i = 10$ (left column) and where $i = 50$ (right column). For the sub-figures in the second row (number of collaborators), the x-axes have been truncated to exclude outliers. Each subfigure has been annotated with its corresponding Chi-Squared test results, all of which were significant ($p < 0.0001$).

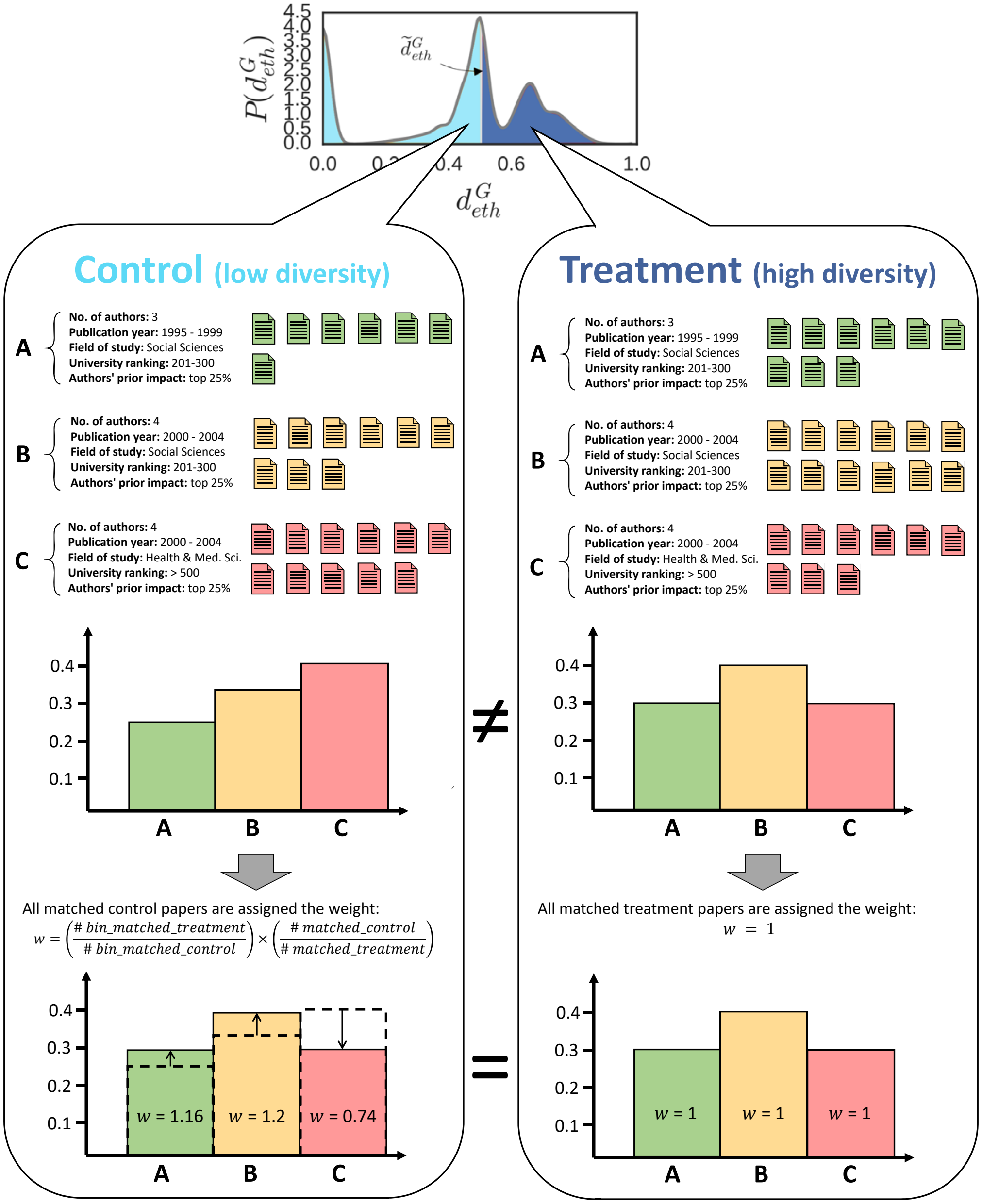

Supplementary Figure 14: An illustration of coarsened exact matching (CEM). The treatment set consists of papers for which $d^G_{eth} > P_{100-i}\left(d^G_{eth}\right)$, and the control set of papers for which $d^G_{eth} \leq P_i\left(d^G_{eth}\right)$, where $P_i\left(d^G_{eth}\right)$ denotes the ith percentile of d^G_{eth}. In our study, we repeated this process using $i = 10, 20, 30, 40, 50$, but in this figure we only illustrate the case where $i = 50$.

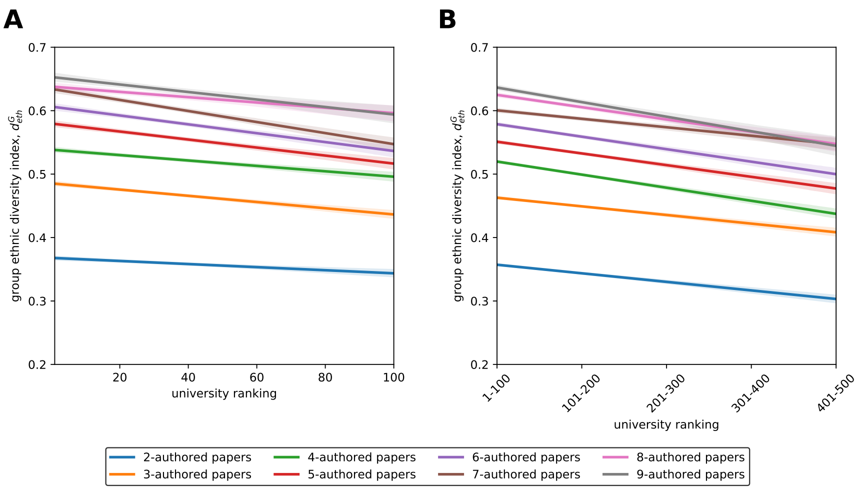

Supplementary Figure 15: University ranking against group ethnic diversity. **(A)** d^G_{eth} for universities ranked $1, 2, \ldots, 100$. **(B)** d^G_{eth} for universities whose ranking falls in one of 5 bins: 1-100; 101-200; 201-300; 301-400; 401-500. In both subfigures, we control for the number of authors per paper. A significant negative correlation between d^G_{eth} and university ranking is found in all cases ($p < 0.001$).

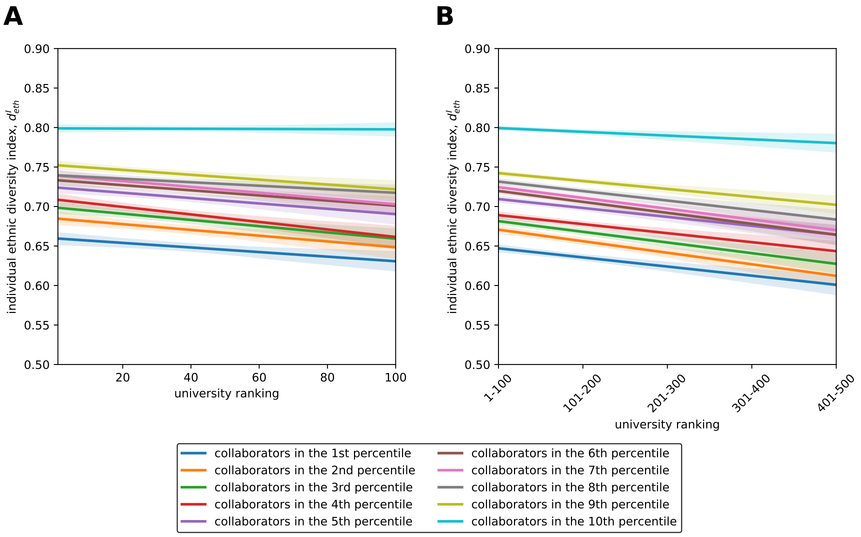

Supplementary Figure 16: University ranking against individual ethnic diversity. **(A)** d^I_{eth} for universities ranked $1, 2, \ldots, 100$. **(B)** d^I_{eth} for universities whose ranking falls in one of 5 bins: 1-100; 101-200; 201-300; 301-400; 401-500. In both subfigures, we control for the number of collaborators per scientist. A significant negative correlation between d^I_{eth} and university ranking is found in all cases ($p < 0.001$).

Supplementary Tables

Supplementary Table 1: Summary of main notation

Notation	Description
S	The set of scientists under consideration
P	The set of papers under consideration
$Papers(s_i)$	The set of papers of scientist s_i
$Authors(p_j)$	The set of authors of paper p_j
$Keywords(p_j)$	The set of keywords in paper p_j
$Gini(M)$	*Gini impurity* of multiset M; see Equation (4)
$Disciplines$	The set of 19 scientific disciplines in *Microsoft Academic Graph (MAG)*
$dsp(s_i)$	Discipline of scientist s_i according to *Microsoft Academic Graph (MAG)*; see Equation (2)
$eth(s_i)$	Ethnicity of scientist s_i according to the *Name Ethnicity Classifier*
$gen(s_i)$	Gender of scientist s_i according to *Genderize.io*
$age(s_i)$	Academic age of scientist s_i, measured by subtracting the publication year of the first paper of s_i from the year 2009
$aff(s_i, p_j)$	Affiliation of scientist s_i in paper p_j according to *Microsoft Academic Graph*
d^G_{dsp}	Group discipline diversity index; see Equation (5), where $x = dsp$
d^G_{eth}	Group ethnic diversity index; see Equation (5), where $x = eth$
d^G_{gen}	Group gender diversity index; see Equation (5), where $x = gen$
d^G_{age}	Group age diversity index; see Equation (5), where $x = age$
d^G_{aff}	Group affiliation diversity index; see Equation (5), where $x = aff$
$\langle d^G_x \rangle$	Average d^G_x taken over a set of papers, where $x \in \{eth, gen, age, dsp, aff\}$
d^I_{dsp}	Individual discipline diversity index; see Equation (6), where $x = dsp$
d^I_{eth}	Individual ethnic diversity index; see Equation (6), where $x = eth$
d^I_{gen}	Individual gender diversity index; see Equation (6), where $x = gen$
d^I_{age}	Individual age diversity index; see Equation (6), where $x = age$
d^I_{aff}	Individual affiliation diversity index; see Equation (6), where $x = aff$
$\langle d^I_x \rangle$	Average d^I_x taken over a set of individuals, where $x \in \{eth, gen, age, dsp, aff\}$
$d^I_x(p_j)$	Average of d^I_{eth} over all authors of p_j, where $x \in \{eth, gen, age, dsp, aff\}$; see Equation (7)
$\left\langle d^I_x \right\rangle_{\text{paper}}$	An alternative notation of $d^I_x(p_j)$ which is often used when the paper p_j is clear from the context
$c^G_5(p_j)$	Number of citations that paper p_j accumulates 5 years after publication
$\langle c^G_5 \rangle$	Average c^G_5 taken over a set of papers
$c^I_5(s_i)$	Number of citations that scientist s_i accumulates on average from his/her papers 5 years after publication; see Equation (8)
$\langle c^I_5 \rangle$	Average c^I_5 taken over a set of individuals
$P_i\left(d^G_{eth}\right)$	The ith percentile of d^G_{eth}

Supplementary Table 2: Pearson's r and p values corresponding to each subfield in Supplementary Figure 8.

Field	r_{eth}	p_{eth}	r_{age}	p_{age}	r_{dsp}	p_{dsp}	r_{aff}	p_{aff}	r_{gen}	p_{gen}
Sustainable Energy	0.06	0.00	0.06	0.00	0.09	0.00	-0.05	0.00	-0.01	0.14†
Mechanical Engineering	0.06	0.00	0.04	0.00	0.04	0.00	-0.06	0.00	-0.02	0.04
Bioinformatics	0.01	0.56†	0.02	0.00	0.02	0.00	0.00	0.68†	-0.03	0.00
Cardiology	0.05	0.00	0.08	0.00	0.04	0.00	0.02	0.01	-0.01	0.00
Psychiatry	0.03	0.00	0.24	0.00	0.05	0.00	-0.02	0.12†	0.05	0.00
Nursing	0.04	0.00	0.02	0.00	0.04	0.00	-0.02	0.12†	-0.00	0.93†
Accounting	0.07	0.00	0.03	0.02	-0.02	0.26†	-0.02	0.44†	-0.01	0.66†
Marketing	0.05	0.03	0.02	0.00	0.02	0.06†	0.02	0.51†	-0.00	0.92†
Educational Administration	0.20	0.00	0.07	0.00	0.07	0.00	0.06	0.04	0.04	0.05
Language & Linguistics	0.07	0.00	0.07	0.00	0.03	0.02	0.02	0.47†	0.06	0.00
Philosophy	0.08	0.03	0.07	0.00	0.03	0.03	-0.04	0.21†	0.00	0.84†
Drama	0.00	0.35†	0.22	0.00	-0.14	0.00	0.01	0.90†	0.04	0.51†
Mathematical Optimization	0.07	0.00	0.02	0.02	0.03	0.00	0.00	0.73†	-0.00	0.80†
Fluid Mechanics	0.02	0.02	-0.01	0.18†	0.02	0.00	-0.02	0.01	-0.04	0.00
Mathematical Physics	0.03	0.00	0.02	0.00	0.01	0.15†	0.04	0.00	-0.04	0.00
Political Science	0.05	0.00	0.08	0.00	0.05	0.00	-0.04	0.03	-0.01	0.47†
Sociology	0.03	0.22†	0.04	0.00	-0.01	0.23†	-0.02	0.34†	0.04	0.00
History	0.20	0.00	0.04	0.00	0.05	0.00	-0.04	0.17†	0.01	0.14†
Oil, Petroleum & Nat. Gas	0.15	0.00	0.05	0.00	-0.07	0.00	0.14	0.00	-0.02	0.30†
Nanotechnology	0.11	0.00	0.05	0.00	0.06	0.00	-0.02	0.12†	-0.01	0.22†
Materials Engineering	0.02	0.00	0.02	0.00	0.05	0.00	0.00	0.76†	-0.01	0.04
Geology	0.04	0.00	0.00	0.65†	0.06	0.00	0.02	0.14†	-0.03	0.00
Environmental Sciences	0.05	0.00	0.04	0.00	0.05	0.00	0.01	0.22†	-0.01	0.00
Ecology	0.01	0.29†	0.06	0.00	0.02	0.00	-0.00	0.84†	-0.02	0.00

† $p \geq 0.05$

Supplementary Table 3: MAG subset definitions. The total number of papers considered is: $9,472,439$ (those include the $1,045,401$ papers in the dataset $\mathcal{D}$). In contrast, the total number of scientists considered is around 6 million: $(1,529,279) + (5,103,877) -$ (overlap between the two sets).

Dataset	Filter	Set Size
Main dataset ($\mathcal{D}$). This is the dataset that will be used by default in all analyses unless stated otherwise.	Of all the papers in the Microsoft Academic Graph (MAG) dataset, we considered all papers from the top five journals from 3 randomly selected subfields from each of the 8 main fields. Then, we removed single-authored and review papers, controlled for English speaking countries, and only retained papers published between 1958 and 2009.	1,045,401 papers, authored by 1,529,279 unique authors.
Dataset to measure group gender diversity index, d^G_{gen}	Papers in $\mathcal{D}$ where the gender of all authors are known with 90% certainty (using Genderize.io).	460,238 papers
Dataset to measure group discipline diversity index, d^G_{dsp}	Papers in $\mathcal{D}$ where the discipline of all authors in a paper is clear and known (i.e. not interdisciplinary) was used.	568,269 papers
Dataset to measure group affiliation diversity index, d^G_{aff}	Papers in $\mathcal{D}$ where each author has exactly one affiliation	207,899 papers
Dataset to measure the individual diversity indices, d^I_x	Scientists in $\mathcal{D}$ with at least 10 collaborators	766,338 scientists with a total of 5,103,877 collaborators taken from 9,472,439 papers

Supplementary Table 4: Pearson's r and p values corresponding to each subfield in Supplementary Figure 10.

Field	r_{eth}	p_{eth}	r_{age}	p_{age}	r_{dsp}	p_{dsp}	r_{aff}	p_{aff}	r_{gen}	p_{gen}
Sustainable Energy	0.05	0.00	0.04	0.00	-0.02	0.55†	-0.03	0.51†	0.00	0.50†
Mechanical Engineering	0.06	0.00	0.07	0.00	0.01	0.79†	-0.06	0.15†	0.00	0.39†
Bioinformatics	0.02	0.00	0.01	0.00	-0.02	0.23†	-0.16	0.00	-0.00	0.01
Cardiology	0.06	0.00	0.05	0.00	0.05	0.12†	0.07	0.00	0.00	0.75†
Psychiatry	0.03	0.00	0.04	0.00	0.05	0.37†	0.10	0.08†	0.02	0.00
Nursing	0.03	0.00	0.04	0.00	0.06	0.32†	-0.34	0.00	0.02	0.00
Accounting	0.06	0.00	0.04	0.00	-0.01	0.86†	0.03	0.61†	-0.02	0.11†
Marketing	0.01	0.05	0.04	0.00	0.12	0.13†	0.10	0.28†	0.00	0.81†
Educational Administration	0.10	0.00	0.06	0.00	0.23	0.00	-0.29	0.00	0.05	0.00
Language & Linguistics	0.07	0.00	0.08	0.00	0.14	0.20†	0.10	0.34†	0.03	0.01
Philosophy	0.06	0.00	0.03	0.08†	0.27	0.04	-0.34	0.00	0.01	0.75†
Drama	0.13	0.00	0.11	0.05†	0.07	0.70†	0.02	0.91†	0.02	0.79†
Mathematical Optimization	0.06	0.00	0.05	0.00	0.07	0.20†	-0.09	0.07†	-0.00	0.62†
Fluid Mechanics	0.06	0.00	0.04	0.00	0.07	0.26†	-0.04	0.53†	-0.01	0.00
Mathematical Physics	0.04	0.00	0.01	0.00	0.04	0.42†	0.07	0.17†	-0.01	0.00
Political Science	0.08	0.00	0.06	0.00	0.25	0.02	-0.14	0.21†	-0.01	0.65†
Sociology	0.06	0.00	0.04	0.00	0.05	0.67†	0.09	0.42†	0.03	0.02
History	0.04	0.00	0.07	0.00	-0.03	0.88†	-0.40	0.29†	0.01	0.25†
Oil, Petroleum & Nat. Gas	0.14	0.00	0.10	0.00	0.02	0.78†	0.16	0.02	-0.03	0.00
Nanotechnology	0.06	0.00	0.04	0.00	0.09	0.13†	0.01	0.89†	0.01	0.00
Materials Engineering	0.02	0.00	0.02	0.00	-0.05	0.32†	0.05	0.29†	0.01	0.00
Geology	0.03	0.00	0.06	0.00	0.03	0.57†	0.09	0.15†	0.03	0.00
Environmental Sciences	0.08	0.00	0.07	0.00	-0.04	0.51†	0.09	0.19†	-0.01	0.00
Ecology	0.04	0.00	0.05	0.00	0.11	0.05†	0.07	0.19†	-0.00	0.90†

† $p \geq 0.05$

Supplementary Table 5: Results of coarsened exact matching on group ethnic diversity. T and C are the treatment and control populations respectively; T' and C' are the populations of matched treatment and matched control papers respectively; $\mathcal{L}_1$ is the multivariate imbalance statistic (*57*); δ is the relative impact gain of T' over C', i.e., $\delta = 100 \times (\langle c_5^G \rangle_{T'} - \langle c_5^G \rangle_{C'}) / \langle c_5^G \rangle_{C'}$. A t-test shows that δ is statistically significant; see the resulting p-values. Since the academic impact $\langle c_5^G \rangle$ is sensitive to extremal values, we bootstrap a 95% confidence interval ($CI_{.95}$). Note that the confounding factor "university ranking" corresponds to the *highest ranked* of all universities in the paper, as opposed to the *average rank* for all universities in the paper, as is the case in Table 2. For more details, see Supplementary Note 5.

	$\|T\|$	$\|C\|$	$\|T'\|$	$\|C'\|$	$\mathcal{L}_1$	δ	$CI_{.95}$	p
$T: d_{eth}^G > P_{90}(d_{eth}^G)$ $C: d_{eth}^G \leq P_{10}(d_{eth}^G)$	17,802	45,710	14,876	16,180	0.38	11.33	[9.12, 13.45]	0.002
$T: d_{eth}^G > P_{80}(d_{eth}^G)$ $C: d_{eth}^G \leq P_{20}(d_{eth}^G)$	24,827	45,710	20,808	16,294	0.38	11.56	[9.52, 13.39]	0.0001
$T: d_{eth}^G > P_{70}(d_{eth}^G)$ $C: d_{eth}^G \leq P_{30}(d_{eth}^G)$	56,662	58,889	53,588	39,376	0.25	5.58	[4.23, 6.75]	0.0066
$T: d_{eth}^G > P_{60}(d_{eth}^G)$ $C: d_{eth}^G \leq P_{40}(d_{eth}^G)$	63,129	78,340	58,903	58,411	0.28	6.50	[5.48, 7.47]	0.0002
$T: d_{eth}^G > P_{50}(d_{eth}^G)$ $C: d_{eth}^G \leq P_{50}(d_{eth}^G)$	63,129	127,629	59,474	70,958	0.26	3.86	[2.96, 4.21]	0.042

Supplementary Notes

Supplementary Note 1. The Data

The Collaboration Network

The data used for this study was obtained on October 2015 from the Microsoft Academic Graph (MAG) database.[1] This is a dataset consisting of scientific publications, their citation records, date of publication, information regarding the authorship (such as name and affiliation), publication venue and more. The dataset also contains a citation network in which every node represents a paper and every directed link represents a citation. While the number of citations of any given paper is not provided explicitly by the dataset, it can easily be calculated from the citation network. More important, the dataset specifies the keywords in each paper, as well as the position of each such keyword in a field-of-study hierarchy, the highest level of which is comprised of 19 disciplines.[2]

Unfortunately, the dataset suffers from three limitations: (i) it does not specify the publication venue's field of science; (ii) it does not specify the *ethnicity* of each scientist; and (iii) it does not specify the *gender* of each scientist. In the following three Supplementary Notes, we show how to overcome these limitations.

Acquiring the Field of Science of Each Publication Venue

To address limitation (i) of Microsoft Academic Graph, we refer to Google Scholar Metrics.[3] Here, journals are categorized into 8 main fields of science, and each such field is divided into multiple subfields. A list of the top 20 publication venues are listed for each subfield. We considered five top journals from 3 randomly selected subfields from each of the 8 main fields.

[1] `https://www.microsoft.com/en-us/research/project/microsoft-academic-graph/`

[2] Note that some keywords fall under multiple disciplines. For instance, according to the dataset, the keyword "Fast fission" has a 50% match with Physics and a 50% match with Chemistry.

[3] `https://scholar.google.com/citations?view_op=top_venues`

The main fields and their subfields are as follows:

1. **Engineering & Computer Science**
   (a) Mechanical Engineering
   (b) Sustainable Energy
   (c) Bioinformatics
2. **Health & Medical Sciences**
   (a) Cardiology
   (b) Psychiatry
   (c) Nursing
3. **Business, Economics & Management**
   (a) Accounting
   (b) Marketing
   (c) Educational Administration
4. **Humanities, Literature & Arts**
   (a) Language & Linguistics
   (b) Drama
   (c) Philosophy
5. **Physics & Mathematics**
   (a) Mathematical Optimization
   (b) Mathematical Physics
   (c) Fluid Mechanics
6. **Social Sciences**
   (a) Political Science
   (b) Sociology
   (c) History
7. **Chemical & Material Sciences**
   (a) Oil, Petroleum & Natural Gas
   (b) Nanotechnology
   (c) Material Engineering
8. **Life Sciences & Earth Sciences**
   (a) Environmental Sciences
   (b) Geology
   (c) Ecology

For each journal, we extracted the data on all papers published therein, and applied the following five filtering steps: (i) removed all single-authored papers (because we are interested in studying collaborations), (ii) controlled for English speaking countries (explained further in section Controlling for Countries), (iii) removed review papers (explained further in section Excluding Review Papers), (iv) extracted data only up to 2009 (the reason behind this is explained in Supplementary Note 4), and (v) only retained papers published in or after 1958 (to deal with missing data issues; in all preceding years, there were multiple fields with no publications at all).

This yielded our main dataset, consisting of 1,045,401 papers, authored by 1,529,279 unique authors. This dataset will be filtered whenever necessary, as will be specified in the following sections (Supplementary Table 3 summarizes all the filters used in our study).

Lastly, to avoid any potential confusion between a scientist's area of science, and a paper's

area of science, we will use the term "discipline" when referring to the former, and use the term "field" when referring to the latter.

Classifying the Ethnicity of Each Scientist

To address limitation (ii) of Microsoft Academic Graph, we used the *Name Ethnicity Classifier*[4] (*38, 39*) to identify the ethnicity of each scientist. In particular, this classifier uses various machine-learning techniques to classify any given name into the following 13 ethnic groups (any unresolved names are marked as "unknown"):

1. Asian, Greater East Asian, East Asian (or "*East Asian*" for short);
2. Asian, Greater East Asian, Japanese (or "*Japanese*" for short);
3. Asian, Indian Sub-Continent (or "*Indian Sub-Continent*" for short);
4. Greater African, Africans (or "*Africans*" for short);
5. Greater African, Muslim (or "*Muslim*" for short);
6. Greater European, British (or "*British*" for short);
7. Greater European, East European (or "*East European*" for short);
8. Greater European, Jewish (or "*Jewish*" for short);
9. Greater European, West European, French (or "*French*" for short);
10. Greater European, West European, Germanic (or "*Germanic*" for short);
11. Greater European, West European, Hispanic (or "*Hispanic*" for short);
12. Greater European, West European, Italian (or "*Italian*" for short);
13. Greater European, West European, Nordic (or "*Nordic*" for short).

As can be seen, the term "ethnicity" is used here in its broader sense, where an ethnic group is defined as *"a social group that shares a common and distinctive culture, religion, language,*

[4] `http://www.textmap.com/ethnicity/`

or the like".[5] The Name Ethnicity Classifier (*38–40*) has an overall accuracy[6] of 80%, which is quite impressive given that the classifier depends solely on the individual's name. Importantly, this accuracy is measured over 20 million distinct names, comprising over 100 million individual entity references (*38*). Admittedly, an accuracy of 80% means that some names will be misclassified. Nevertheless, unlike conventional methods where ethnicity is identified manually, this classifier allows for conducting studies at an unprecedented scale, e.g., involving millions of names. In our case, we were able to obtain the ethnicity of every single scientist in our study.

Identifying the Gender of Each Scientist

To address limitation (iii) of Microsoft Academic Graph, we needed to identify the gender of each scientist in our dataset. To this end, a number of alternative methods have been proposed in the literature to identify the gender (either male or female) of any given individual based solely on his/her first name (*41–43*). Out of all those alternatives, a software tool called "'*Genderize.io*", which is available at: `https://genderize.io/`, was shown to be the most reliable (*41*). Note that gender identification based solely on first name is indeed very challenging (if not impossible, in some cases), due mainly to the fact that some names are unisex. As such, it is perhaps not surprising that 47.71% of the names tested on Genderize.io were unclassified, i.e., the tool returned "unknown" for each such name (*41*). Nevertheless, this tool outperformed the alternative methods, for which the number of unclassified names exceeded 84%. As for the names that were classified, the "error rate" represents the percentage of names whose classification was incorrect. While the alternative methods had an error rate greater that 32%, Genderize.io had an error rate of just 7%, which is impressive given that the input to this

[5] http://www.dictionary.com/browse/ethnicity?s=t

[6] While the authors report the results of each ethnicity independently, the overall accuracy can easily be computed from these results.

gender-identification tool is merely the first name of the individual in question. Admittedly, an error rate of 7% means that some genders will be misclassified. Nevertheless, unlike conventional methods where gender is identified manually, this automated tool allows for conducting studies at an unprecedented scale, covering thousands, or even millions of names. Using Genderize.io, we were able to classify the gender of 3,183,911 scientists. To further increase our confidence of the gender classification, we considered only the 2,046,359 names that were classified with at least 90% confidence.

Controlling for Countries

To control for countries, we consider papers of which the majority of the authors' affiliations belong to any of the following countries: USA, UK, Canada and Australia. The rationale behind this choice is threefold:

1. These are predominantly English-speaking countries.[7] As English is widely considered the universal language of science,[8] limiting our study to English-speaking countries should cover a wide range of cultural, ethnic and national backgrounds.

2. These countries have ethnically diverse populations and higher-education systems that attract large numbers of international students and faculty members. In contrast, universities in many other countries (such as Japan and China) have student populations of which the vast majority are of a single ethnic group.

3. Universities from those four countries form a significant proportion of the world's leading institutions (as reported in the Times Higher Education 2018 rankings, 95% of the world's top 20, and 63% of the top 100 universities are from these four countries[9]).

[7] Although Canada has two official languages, namely English and French, 56.9% of the Canadian population report English as their mother tongue; see: www.statcan.gc.ca.

[8] https://www.researchtrends.com/issue-31-november-2012/the-language-of-future-scientific-communication/

[9] https://www.timeshighereducation.com/world-university-rankings/2018/world-ranking#!/

To put it differently, most papers (co)authored in these four countries are arguably (i) written in the same language, (ii) produced in environments that permit the formation of diverse teams and (iii) relatively more likely to produce high-impact research. The above factors help to ensure that the papers studied are, in general, highly impactful and comparable. Of all the affiliations present in the Microsoft Academic Graph dataset, 5,899 (out of a total of 19,788) were manually verified to be based in one of the aforementioned four countries.

Excluding Review Papers

Review papers exhibit different statistics (*44–46*), and could bias our results. As such, we excluded from our analysis any review papers that we could find based on tell-tale words that could be found in the keywords of the paper, such as "literature review", "literature", or "survey". Following this process, 11,367 review papers were found and removed from our dataset.

Supplementary Note 2. Quantifying Diversity

This note starts by discussing the five classes of diversity that are considered in our study, followed by a discussion of the group and individual diversity indices, respectively. A summary of all notation is provided in Supplementary Table 1.

Classes of Diversity

When exploring diversity in research collaborations, we investigate five classes of diversity:

1. **Ethnic diversity:** This class of diversity takes into consideration the ethnic background of each scientist. As described in Supplementary Note 1, we use the *Name Ethnicity Classifier* to identify the ethnicity of each scientist.

2. **Gender diversity:** This class of diversity takes into consideration the gender of each scientist, which is identified using *Genderize.io*. When studying gender diversity, we only include a paper if the gender of each of its author is identified by *Genderize.io*; see Supplementary Note 1 for more details on how gender is identified, and Supplementary Table 3 for a summary of all datasets used in our study.

3. **Age diversity:** Here, "age" refers to the academic age of a scientist, which is measured in each paper, p, by subtracting the year of the scientist's first paper from the year in which p was published. The resulting dataset is then divided into the following bins:

   - Academic age group 0 : 0-9 years of experience;
   - Academic age group 1 : 10-19 years of experience;
   - Academic age group 2 : 20-29 years of experience;
   - Academic age group 3 : 30-39 years of experience;
   - Academic age group 4 : 40-49 years of experience;
   - Academic age group 5 : $\geqslant$50 years of experience.

4. **Discipline Diversity:** This class of diversity takes into account the co-authors' area of expertise. We determine the discipline of each scientist based on the keywords that are specified in his/her papers. This is made possible by the fact that the MAG dataset specifies the probability of each keyword belonging to any of the following 19 disciplines:

(1) Art
(2) Biology
(3) Business
(4) Computer Science
(5) Chemistry
(6) Economics
(7) Engineering
(8) Environmental science
(9) Geography
(10) Geology
(11) History
(12) Materials Science
(13) Mathematics
(14) Medicine
(15) Philosophy
(16) Physics
(17) Political Science
(18) Psychology
(19) Sociology

Formally, the probability of scientist s_i belonging to discipline x_j is calculated as follows:

$$P(dsp(s_i) = x_j) = \frac{\sum\limits_{p \in Papers(s_i)} \sum\limits_{w \in Keywords(p)} P(dsp(w) = x_j)}{\sum\limits_{x_k \in Disciplines} \sum\limits_{p \in Papers(s_i)} \sum\limits_{w \in Keywords(p)} P(dsp(w) = x_k)} \quad (1)$$

where $Papers(s_i)$ denotes the set of papers of scientist s_i, $Keywords(p)$ denotes the set of keywords of paper p, $P(dsp(w) = x_j)$ denotes the probability that the keyword w belongs to the discipline x_j, and $Disciplines$ denotes the set of the 19 disciplines in MAG. Then, the discipline of scientist s_i is determined as follows:

$$dsp(s_i) = \begin{cases} \arg\max\limits_{x_k \in Disciplines} P(dsp(s_i) = x_k) & \text{if } \max\limits_{x_k \in Disciplines} P(dsp(s_i) = x_k) > 0.5 \\ \text{“unknown”} & \text{if } \max\limits_{x_k \in Disciplines} P(dsp(s_i) = x_k) \leq 0.5 \end{cases} \quad (2)$$

where $P(dsp(s_i) = x_k)$ is calculated as in Equation (1). We exclude from our analysis any paper of which the discipline of an author is "unknown" (see Supplementary Table 3 for a summary of all the filters applied on our dataset).

5. **Affiliation Diversity:** This class of diversity takes into consideration the affiliations of the co-authors of a paper. Note that, where available, MAG specifies the affiliation of every author on every paper. As such, a scientist's affiliation may vary from one paper to another. We exclude any papers where an author has more than one affiliation or no affiliation at all. This way, having multiple affiliations on a paper indicates that it is the result of collaboration across different research entities (see Supplementary Table 3).

Measuring Diversity

The diversity of any given group reflects the degree to which its members differ from one another. To study the relationship between this property and the success of the associated group, a numerical measure of group diversity is required. To this end, several metrics have been proposed, the majority of which fall into two main categories:

1. Metrics that measure diversity by quantifying the uncertainty in predicting the type of an element drawn randomly from the set in question. Such a metric is commonly known as the *Shannon entropy* or the *Shannon-Wiener Index*. Formally, given k types, and a set S, the Shannon entropy is computed as in Equation (3), where $p_i(S)$ denotes the proportion of the elements of S that are of the ith type.

$$Shannon(S) = -\sum_{i=1}^{k} p_i(S) \ln p_i(S). \tag{3}$$

2. Metrics that are designed to reflect the degree of concentration when the group members are classified into types (*47*). Such a metric is commonly known as the *Simpson index* in ecological literature, and as the *Herfindahl-Hirschman index* in the economic literature

(*48*). It can also be found, with slight variations, in other fields under different names, including the *probability of interspecific encounter* (*49*), the *Gini-Simpson index* (*50*), and the *Gini impurity* (*51*). The formula for the Gini impurity is computed as shown in Equation (4).

For every paper in the entire MAG dataset, we measured the ethnic diversity in the group of authors using the Shannon entropy and using the Gini impurity. The two measures are plotted against each other in Supplementary Figure 2. As can be seen, the two are strongly correlated, with Pearson's $r = 0.93$ and $p < 0.0001$. Based on this, throughout the remainder of our study, we focus on just one of those measures, namely the Gini impurity, which will be explained in more detail in the following section.

Group Diversity Index

In this section, we explain how the *Gini impurity* (*52*) is used to measure the diversity in any given paper. To this end, we need to introduce some additional notation. Let S and P denote the set of scientists and the set of papers under consideration, respectively. Furthermore, let $Authors(p_j) \subseteq S$ denote the set of authors of paper p_j. Now, for any given scientist $s_i \in S$, let $eth(s_i)$, $gen(s_i)$, $dsp(s_i)$, and $age(s_i)$ denote the *ethnicity*, the *gender*, the *discipline* and the *academic age* of s_i, respectively. Similarly, let $\mathit{aff}(s_i, p_j)$ denote the *affiliation* of scientist s_i on paper p_j.[10] For details on how the ethnicity, gender, discipline, academic age, and affiliation are identified, section above on Classes of Diversity. Note that for any given paper, p_j, any set $\{x(s_i) : s_i \in Authors(p_j)\}$ such that $x \in \{eth, gen, age, dsp\}$ is actually a multiset. Likewise, the set $\{\mathit{aff}(s_i, p_j) : s_i \in Authors(p_j)\}$ is also a multiset. When dealing with multisets, we will use square brackets instead of curly ones. For instance, for any given paper, p_j, we could have: $[eth(s_i) : s_i \in Authors(p_j)] = [\text{Japanese}, \text{British}, \text{British}]$,

[10]The affiliation of s_i is denoted by $\mathit{aff}(s_i, p_j)$ rather than $\mathit{aff}(s_i)$ because the affiliation of a scientist may vary from one paper to another.

and have: $[\mathit{aff}(s_i, p_j) : s_i \in \mathit{Authors}(p_j)] = [\text{Harvard}, \text{Harvard}, \text{Stanford}]$. For any given multiset, M, let $|M|$ denote the cardinality of M, let $\mathit{under}(M)$ denote the underlying set of M, and let $\mathit{multi}(m, M)$ denote the multiplicity of element m in M. For example, given $M = [\text{Harvard}, \text{Harvard}, \text{Stanford}]$, we have: $|M| = 3$, $\mathit{under}(M) = \{\text{Harvard}, \text{Stanford}\}$, $\mathit{multi}(\text{Harvard}, M) = 2$ and $\mathit{multi}(\text{Stanford}, M) = 1$. The ***Gini impurity*** of a multiset, M, is then defined as:

$$\mathit{Gini}(M) = 1 - \sum_{m \in \mathit{under}(M)} \mathit{proportion}(m, M)^2, \tag{4}$$

where

$$\mathit{proportion}(m, M) = \frac{\mathit{multi}(m, M)}{|M|}.$$

With this notation in place, we are now ready to formally define our *group diversity index*. In particular, for any given paper, $p_j \in P$, the group diversity index of p_j is defined as follows, where the "G" in d_x^G stands for "***Group***":

$$d_x^G(p_j) = \begin{cases} \mathit{Gini}\left([x(s_i) : s_i \in \mathit{Authors}(p_j)]\right) & \text{if } x \in \{\mathit{eth}, \mathit{gen}, \mathit{dsp}, \mathit{age}\} \\ \mathit{Gini}\left([x(s_i, p_j) : s_i \in \mathit{Authors}(p_j)]\right) & \text{if } x = \mathit{aff} \end{cases} \tag{5}$$

We will often omit the paper, p_j, from the notation $d_x^G(p_j)$ and simply write d_x^G whenever the paper itself is clear from the context.

Next, we summarize our five group diversity indices, and specify the papers that were considered for each such index (out of all 1,045,401 papers published in our dataset):

1. d_{eth}^G—the "*group ethnic diversity index*"; we calculated this for all papers in our dataset.

2. d_{gen}^G—the "*group gender diversity index*"; for any paper, we calculate this index only if the gender of each of author has been identified by Genderize.io.

3. d_{age}^G—the "*group age diversity index*"; this was calculated for all papers in our dataset.

4. d^G_{dsp}—the "*group discipline diversity index*"; for this index, we exclude every paper of which an author's discipline is "unknown" according to Equation (2).

5. d^G_{aff}—the "*group affiliation diversity index*"; we calculated this index for every paper whose authors each have exactly one affiliation on the paper (i.e., we exclude papers of which an author has more than one affiliation, or no affiliation at all).

Individual Diversity Index

For any given scientist, $s_i \in S$, the *individual diversity index* of s_i is defined as follows:

$$d^I_x(s_i) = \begin{cases} \mathit{Gini}\left(\biguplus_{p_j \in \mathit{Papers}(s_i)} \big[x(s_k) : s_k \in \mathit{Authors}(p_j) \setminus \{s_i\}\big]\right) & \text{if } x \in \{\mathit{eth}, \mathit{gen}, \mathit{dsp}, \mathit{age}\} \\ \mathit{Gini}\left(\biguplus_{p_j \in \mathit{Papers}(s_i)} \big[x(s_k, p_j) : s_j \in \mathit{Authors}(p_j) \setminus \{s_i\}\big]\right) & \text{if } x = \mathit{aff} \end{cases} \tag{6}$$

where "I" in d^I_x stands for "*Individual*", $\mathit{Papers}(s_i)$ denotes the set of papers of which scientist s_i is an author, $\uplus$ denotes the multiset sum operation, and Gini is defined as in Equation (4). We will clarify the notation through an example. Suppose that scientist A is an author of just two papers, p_1 and p_2, such that:

- $\mathit{Authors}(p_1) = \{A, B, C\}$;
- $\mathit{Authors}(p_2) = \{A, C, D\}$;
- the ethnicities of B, C, and D are Japanese, British, and French, respectively.

Then we would have:

$$\begin{aligned} \biguplus_{p_j \in \mathit{Papers}(A)} \big[\mathit{eth}(s_k) : s_k \in \mathit{Authors}(p_j) \setminus \{A\}\big] &= [\mathit{eth}(B), \mathit{eth}(C)] \uplus [\mathit{eth}(C), \mathit{eth}(D)] \\ &= [\text{Japanese, British}] \uplus [\text{British, French}] \\ &= [\text{Japanese, British, British, French}]. \end{aligned}$$

We will overload the notation by letting $d_x^I(p_i)$ denote the average individual diversity of the authors of paper p_i. More formally:

$$d_x^I(p_i) = \frac{\sum\limits_{s_i \in Authors(p_i)} d_x^I(s_i)}{|Authors(p_i)|}, \tag{7}$$

where $x \in \{eth, gen, age, dsp, aff\}$. To improve readability, we may write $\left\langle d_{eth}^I \right\rangle_{\text{paper}}$ instead of $d_x^I(p_i)$ when p_i is clear from the context. Moreover, when dealing with individual scientists, we will often write d_x^I instead of $d_x^I(s_i)$ when s_i is clear from the context.

To summarize, our five individual diversity indices are as follows:

1. d_{eth}^I—the "*individual ethnic diversity index*";
2. d_{gen}^I—the "*individual gender diversity index*";
3. d_{age}^I—the "*individual age diversity index*";
4. d_{dsp}^I—the "*individual discipline diversity index*";
5. d_{aff}^I—the "*individual affiliation diversity index*".

Out of the 1,529,279 scientists in our dataset, we calculated the individual diversity index for those with at least ten collaborators each; this yielded a total of 766,338 scientists with 5,103,877 collaborators taken from 9,472,439 different papers. Furthermore, when studying the average individual diversity in each subfield, we excluded any scientist whose name appears in more than one subfield in our dataset. This led to the exclusion of 6.8% of the scientists . For a summary of all filters applied on our dataset, see Supplementary Table 3.

Supplementary Note 3. The Randomized Baseline Model

In an attempt to isolate the effect of diversity from other confounding factors, we analyzed a randomized baseline model in which the scientists' ethnicities are shuffled while preserving all other characteristics. To explain how such a model is generated, we need some additional notation. For every paper, $p_j \in P$, let $Field(p_j)$ denote the subfield of p_j, and let $Year(p_j)$ denote the publication year of p_j. Furthermore, for every scientific subfield, f, number of authors, n, and publication year, y, let us denote by $S_{f,n,y} \subset S$ the set consisting of every author of a paper in subfield f with n authors, published in year y. More formally:

$$S_{f,n,y} = \bigcup_{p_j \in P : Field(p_j)=f, |Authors(p_j)|=n, Year(p_j)=y} \{s \in Authors(p_j)\}.$$

With this notation in place, we are ready to describe how the randomized baseline model is generated. To improve readability, our description will focus on ethnic diversity; for the other classes of diversity, simply replace eth with the class of choice.

Recall that $eth(s)$ denotes the ethnicity of scientist s. Then, for each scientific subfield, f, number of authors, n, and publication year, y, the ethnicities in $S_{f,n,y}$ are shuffled as follows:

1. Create a list, $L_{f,n,y}$, such that $L_{f,n,y}[i] := eth(s_i), \forall s_i \in S_{f,n,y}$;

2. Create a list, $L'_{f,n,y}$, which is a shuffled version of $L_{f,n,y}$;

3. Set the ethnicities in the randomized model as follows: $eth(s_i) := L'_{f,n,y}[i], \forall s_i \in S_{f,n,y}$.

The entire process was repeated 1,000 times, and the average *group* ethnic diversity, d^G_{eth}, and average *individual* ethnic diversity, d^I_{eth}, were used.

Supplementary Note 4 Scientific Impact: Citation Counts

In their expansive study on scientific impact, Sinatra et al. (*53*) studied the number of citations that a paper accumulates 10 years after publication, denoted by c_{10}; the same impact measure was later on used in (*54*). We follow a similar approach, but focus on 5 rather than 10 years. This way, we incorporate more recent papers in our study, which is particularly important since the majority of the papers in our study were published in recent years (Supplementary Figure 1). Based on this, as well as the fact that our dataset was obtained in October 2015, we only calculate c_5 for papers published between 1958 and 2009.

We distinguish between the number of citations that a *paper* accumulates, and the number of citations that a *scientist* accumulates. To this end, we introduce the following notation:

1. $c_5^G(p_j)$: The number of citations that paper p_j accumulates 5 years after publication, where "G" stands for "Group";

2. $c_5^I(s_i)$: The average number of citations that scientist s_i accumulates from a paper 5 years after its publication, where "I" stands for "Individual". More formally:

$$c_5^I(s_i) = \frac{\sum\limits_{p_j \in Papers(s_i)} c_5^G(p_j)}{|Papers(s_i)|}. \tag{8}$$

To improve readability, we will often write c_5^G instead of $c_5^G(p_j)$ whenever the paper is clear from the context. Similarly, we will write c_5^I instead of $c_5^I(s_i)$ when there is no risk of confusion.

Various studies have demonstrated that the average number of citations per paper changes over time (*44, 53, 55, 56*). To mitigate this temporal effect, we follow the approach proposed by Sinatra et al. (*53*), and consider an alternative, normalized measure of impact, defined as:

$$\widetilde{c_5^G} = \frac{c_5^G(p_j)}{\left\langle c_5^G \right\rangle_{year(p_j)}},$$

where $\langle c_5^G \rangle_{year(p_j)}$ denotes the average c_5 taken over all papers published in the same year as p_j. Similarly, when analyzing the impact of a scientist s_i, we considered an alternative, normalized version of $c_5^I(s_i)$, defined as follows:

$$\widetilde{c}_5^I(s_i) = \frac{\sum\limits_{p_j \in Papers(s_i)} \widetilde{c}_5^G(p_j)}{|Papers(s_i)|}. \tag{9}$$

Considering every paper in the entire MAG dataset, we found that c_5^G and $\widetilde{c}_5^G$ are very strongly correlated, with Pearson's $r = 0.965$ and $p < 0.0001$ (Supplementary Figure 7 depicts c_5^G against $\widetilde{c}_5^G$ for 500,000 papers chosen uniformly at random). Note that there is no need to repeat this analysis for $\widetilde{c}_5^I$ since it is derived from $\widetilde{c}_5^G$; see Equation (9). Based on this finding, all subsequent analysis uses the unnormalized versions, i.e., c_5^G and c_5^I, since they seem to be more intuitive and interpretable, as argued in (*53*).

Supplementary Note 5. Coarsened Exact Matching

To establish a causal link between ethnic diversity and scientific impact, we use *coarsened exact matching (CEM)* (*57*), a technique used to infer causality in observational studies. Specifically, it matches the control and treatment populations with respect to the confounding factors identified, thereby eliminating the effect of these factors on the phenomena under investigation. In our case, when studying a paper's *group* ethnic diversity, we identified the following confounding factors and bins (we experimented with other binning decision and the results were found to be robust to the binning decisions):

- **year of publication**: 5 bins, the first of which contains papers published before 1990; the remaining 4 bins reflect 5-year intervals between 1990 and 2010.
- **number of authors**: Each bin corresponds to a single number.
- **field of study**: 8 bins, one for each of the main fields of science (see Supplementary Note 1).
- **authors' impact prior to publication**: An author's prior impact is measured as the average number of citations that he/she accumulated per year over the period that precedes the year in which the paper was published. The prior impact of all authors is binned into 3 bins, one corresponding to the top 25%, one the middle 50%, and one the lowest 25%.
- **university rankings**:[11] Here, we consider two alternatives:
  - 6 bins, corresponding to the rank of the **highest-ranked** university; this rank falls in one of the following: 1-100; 101-200; 201-300; 301-400; 401-500; >500.
  - 6 bins, corresponding to **average rank** of all universities in the paper; this average falls in one of the following: 1-100; 101-200; 201-300; 301-400; 401-500; >500.

[11]University rankings are based on the 2017 *"Academic Ranking of World Universities"*, also known as the *"Shanghai ranking"*; see `http://www.shanghairanking.com/ARWU2017.html`

In contrast, when studying an author's *individual* ethnic diversity, we identified these confounding factors and bins (we experimented with other binning decisions and got similar results):

- **academic age**: Each bin corresponds to a single academic age.
- **number of collaborators**: Each bin corresponds to a single number.
- **discipline**: 19 bins, one for each discipline (see Supplementary Note 2).
- **university ranking**: 6 bins, corresponding to scientists whose affiliation rank falls in one of the following: 1-100; 101-200; 201-300; 301-400; 401-500; $>$500.

Next, we filter the dataset and retain only papers and scientists for which the above confounding factors are known. Throughout the remaining steps of CEM, we will only deal with this filtered dataset. We now move on to selecting the treatment set, T, and the control sets, C. To this end, let $P_i\left(d^G_{eth}\right)$ be the ith percentile of d^G_{eth}. Then, when studying *group* ethnic diversity, the treatment and control sets consist of papers for which $d^G_{eth} > P_{100-i}\left(d^G_{eth}\right)$, and $d^G_{eth} \leq P_i\left(d^G_{eth}\right)$, respectively, where $P_i\left(d^G_{eth}\right)$ denotes the ith percentile of d^G_{eth}. This process is repeated using $i = 10, 20, 30, 40, 50$, corresponding to progressively larger gaps in ethnic diversity between the two populations. Thus, if ethnic diversity does indeed increase scientific impact, we would expect to find a significant difference in impact between the two populations, and expect the difference to increase in tandem with the aforementioned gap in diversity. The same process was carried out for *individual* ethnic diversity, but with d^I_{eth} instead of d^G_{eth}. This CEM process is illustrated in Supplementary Figure 14, whereas the distributions of the confounding factors in both treatment and control groups (before the CEM process) are depicted in Supplementary Figures 12 and 13.

The CEM results for *group* ethnic diversity can be found in Table 2 of the main article, whereby the confounding factor "university ranking" corresponds to the *average rank* of all universities in the paper. Similarly, the CEM results for *individual* ethnic diversity can be found

in Table 3 of the main article, whereby the “university ranking” corresponds to the rank of the scientist’s affiliation. In contrast, here we present the CEM results for group ethnic diversity whereby “university ranking” corresponds to the rank of the *highest-ranked* university in the paper; see Supplementary Table 5. Similar broad trends can be observed, compared to Tables 2 and 3.

Supplementary Note 6. The Relationship between University Rankings and Ethnic Diversity

In this Supplementary Note, we investigate whether the correlation between ethnic diversity and research impact is due to higher-ranked universities attracting top students with diverse ethnic backgrounds from abroad. University rankings were based on the 2017 “Academic Ranking of World Universities”, also known as the “Shanghai ranking”.[12] Supplementary Figure 15 depicts the following while controlling for the number of authors per paper:

- Group ethnic diversity against the following university rankings: 1, 2, ..., 99, 100;
- Group ethnic diversity against the following university ranking bins: 1-100; 101-200; 201-300; 301-400; 401-500;

In contrast, Supplementary Figure 16 depicts the following while controlling for the number of collaborators per scientist:

- Individual ethnic diversity against the following university rankings: 1, 2, ..., 99, 100;
- Individual ethnic diversity against the following university ranking bins: 1-100; 101-200; 201-300; 301-400; 401-500.

As anticipated, the correlation between ethnic diversity and university ranking is negative and significant in all cases ($p < 0.001$), e.g., a university ranked 10th produces, on average, papers with greater ethnic diversity than another ranked 80th. Nevertheless, even when controlling for university ranking, the relationship between ethnic diversity and scientific impact persists, as was shown in Supplementary Note 5.

[12] http://www.shanghairanking.com/ARWU2017.html

Supplementary References

38. Ambekar, A., Ward, C., Mohammed, J., Male, S. & Skiena, S. Name-ethnicity classification from open sources. In *Proceedings of the 15th ACM SIGKDD international conference on Knowledge Discovery and Data Mining*, 49–58 (ACM, 2009).

39. Ye, J. *et al.* Nationality classification using name embeddings. In *Proceedings of the 2017 ACM on Conference on Information and Knowledge Management*, 1897–1906 (ACM, 2017).

40. Chen, S. A.i. research is in desparate need of an ethical watchdog. *Wired* .

41. Wais, K. Gender prediction methods based on first names with genderizer. *R Journal* **8** (2016).

42. West, J. D., Jacquet, J., King, M. M., Correll, S. J. & Bergstrom, C. T. The role of gender in scholarly authorship. *PloS one* **8**, e66212 (2013).

43. Larivière, V., Ni, C., Gingras, Y., Cronin, B. & Sugimoto, C. R. Bibliometrics: Global gender disparities in science. *Nature News* **504**, 211 (2013).

44. Radicchi, F., Fortunato, S. & Castellano, C. Universality of citation distributions: Toward an objective measure of scientific impact. *Proceedings of the National Academy of Sciences* **105**, 17268–17272 (2008).

45. Radicchi, F. & Castellano, C. Testing the fairness of citation indicators for comparison across scientific domains: The case of fractional citation counts. *Journal of Informetrics* **6**, 121–130 (2012).

46. Stringer, M. J., Sales-Pardo, M. & Amaral, L. A. N. Statistical validation of a global model for the distribution of the ultimate number of citations accrued by papers published in a scientific journal. *Journal of the Association for Information Science and Technology* **61**, 1377–1385 (2010).

47. Simpson, E. H. Measurement of diversity. *nature* **163**, 688 (1949).

48. Herfindahl, O. C. *Concentration in the steel industry*. Ph.D. thesis, Columbia University New York (1950).

49. Hurlbert, S. H. The nonconcept of species diversity: a critique and alternative parameters. *Ecology* **52**, 577–586 (1971).

50. Jost, L. Entropy and diversity. *Oikos* **113**, 363–375 (2006).

51. Rokach, L. & Maimon, O. Top-down induction of decision trees classifiers-a survey. *IEEE Transactions on Systems, Man, and Cybernetics, Part C (Applications and Reviews)* **35**, 476–487 (2005).

52. Bishop, C. M. *Pattern recognition and machine learning* (Springer, 2013).

53. Sinatra, R., Wang, D., Deville, P., Song, C. & Barabási, A.-L. Quantifying the evolution of individual scientific impact. *Science* **354**, aaf5239 (2016).

54. Fortunato, S. *et al.* Science of science. *Science* **359**, eaao0185 (2018).

55. Bornmann, L. & Daniel, H.-D. What do citation counts measure? a review of studies on citing behavior. *Journal of documentation* **64**, 45–80 (2008).

56. Althouse, B. M., West, J. D., Bergstrom, C. T. & Bergstrom, T. Differences in impact factor across fields and over time. *Journal of the Association for Information Science and Technology* **60**, 27–34 (2009).

57. Iacus, S. M., King, G. & Porro, G. Causal inference without balance checking: Coarsened exact matching. *Political analysis* **20**, 1–24 (2012).
